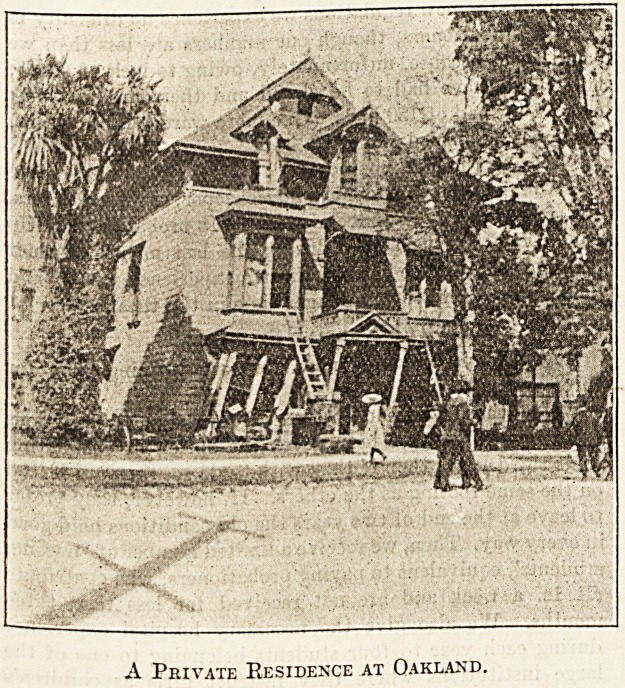# "The Hospital" Nursing Section

**Published:** 1906-05-26

**Authors:** 


### The Hospital.

### Nursing Section.

Contributions for "The Hospital," should be addressed to the Editor, "The Hospital" Nursing Section, 28 & 29 Southampton Street, Strand, London, W.C.

No. 1,026.—Vol. XL. SATURDAY, MAY 26, 1906.

### Notes on News from the Nursing World.

### MATRON APPLICANTS FOR OFFICE.

Candidates for matronships should make it their business to visit the hospital where a vacancy has occurred before they apply for the post. If they know their work such an inspection will reveal much which may be useful to them in forming a judgment as to whether or not they should apply and, if they do apply, as to what they shall say when they come before the committee for election.

### AN ENGLISH NURSE AND THE SAN FRANCISCO DISASTER.

The experiences of an English nurse at the time of the earthquake in San Francisco, which we are able to give in another page, will be read with the greatest interest, and we think that there will be much admiration felt for the pluck and perseverance of our correspondent. The illustrations are made from photographs which were taken eighteen hours after the earthquake, and as the place was then a mass of flame and smoke they are, of course, not very clear. The photographer who took them and gave them to our correspondent to use had a narrow escape. He had just gone into the cupboard to put back his Kodak when the floor above fell in and imprisoned him until he was rescued. Our correspondent apologises because she found it impossible to get her article type-written, as the few typewriters who had a machine left were all overworked.

### AN INSTITUTIONAL BADGE.

The citizens of Salisbury have no difficulty in identifying the nurses attached to the General Infirmary. As the matron informed our Commissioner, in the interview reported elsewhere, the members of the nursing staff, from herself to the probationers, are given and required to wear a badge, upon the bar of which is painted "Salisbury Infirmary." The badges differ in appearance, according to the status of the wearers; but the adoption of an institutional badge seems, in this case, at all events, to command the approval of all parties. It is obvious that no untrained nurse in Salisbury can pass herself off as one of the Infirmary staff. The plan strikes us as worthy of being tried in other hospitals.

### FRESH FRICTION AT EXETER INFIRMARY.

A further conflict has arisen between the nursing staff at Exeter Poor-law Infirmary and the workhouse master, whose interference with details in hospital management is resented. At the meeting on Tuesday of Exeter Guardians a letter was read from the assistant nurses at the new Workhouse Infirmary, stating that the master wished them to apply to him personally for leave of absence. The superintendent nurse had always arranged their time to suit their work; but now, according to the master's new rule, they would have to ask him before they altered their time. The present leave of absence was two hours daily, and every other Sunday from three to ten. Extra time was granted by the superintendent nurse, who reported to the master. The nurses were all satisfied, and wished to keep to this rule. Mr. Surridge moved that the letter be referred to the Management Committee, and Canon Hobson urged that in the meantime no alteration should be made in the rule for nurses. Mrs. Chorley said that the present system had worked satisfactorily, and she could not see why it should be altered. As far as she was concerned she would vote upon the question at once, for she considered it was wrong in the master to interfere with the nurses as he had done. It was ultimately agreed to refer the letter to the Management Committee, and in the meantime no alteration in the nursing rules will be permitted.

### THE EXCHANGE PRINCIPLE.

It will be recollected that some little time ago the authorities of the West Riding of Yorkshire adopted a scheme for the temporary transfer or loan of nurses between isolation hospital managers, with the view of meeting the fluctuating requirements of these institutions. A meeting has lately been held at Gloucester to consider the desirability of exchanging nurses under similar conditions, and it was unanimously resolved by the representatives of county, borough, and hospital authorities present to prepare a scheme for the combined counties of Gloucester and Worcester and the county borough of Bristol. An influential committee was constituted, and they will report as early as possible the result of their deliberations and inquiries. There is no doubt that in the West Riding of Yorkshire the principle has worked well, and we expect that as time goes on it will find increasing favour, both on the grounds of economy and of efficiency. At present it is too soon to judge how far the idea of being "loaned" is popular amongst nurses.

### A PATRIOTIC NURSE.

A handsome donation from a member of the Army Nursing Service Reserve has been acknowledged by the Royal Patriotic Fund Corporation. She has contributed the sum of £700 to the Transvaal War Fund for widows, orphans, and other dependents of officers and men who lost their lives in the South African War, towards which the corporation are still glad to receive assistance. We are afraid that few nurses, with all the will in the world, have the power to make such a contribution as this, but no doubt the donor has been greatly impressed by her personal experiences with the pecuniary distress caused by the war, which remains long after all public interest in it has ceased.

### AN UNDER-STAFFED POOR-LAW INFIRMARY.

At the last meeting of the Knaresborough Guardians it was announced that only three applications had been received in answer to an advertisement for an assistant nurse, and that not one of the candidates was eligible. The conclusion of a lady Guardian was that the salary of £25 a year offered was not enough, and the medical officer stated that the conditions under which the nurses of Knaresborough Workhouse Infirmary worked were not favourable. He considered that the staff was insufficient. It was decided to offer a salary of £26, rising yearly by increments of £2 to £30. But if Knaresborough Infirmary has a bad name because it is understaffed, the difficulty in obtaining nurses is likely to increase rather than to diminish.

### CASH INSTEAD OF UNIFORM.

The Guardians of Wigton, in Cumberland, having selected a probationer, were informed by the applicant appointed that she preferred cash instead of uniform, and at their last meeting they discussed the question. A suggestion was made that the sum of £3 10s. should be offered, but the Chairman and another Guardian favoured £5. Ultimately a motion in support of a grant of £5 was submitted, but an amendment to the effect that it be £3 10s. was carried by 16 votes to 2. We think that it would have been better if the Guardians had informed the probationer that they preferred to adhere to the custom of providing the uniform.

### VACANCIES IN THE ARMY SERVICE.

There are vacancies for staff nurses in Queen Alexandra's Imperial Military Nursing Service, which it is desired to fill forthwith. The salary to begin with is £40, and, under the new rates of pay, there is an annual increase to £45. Applicants must be between the ages of 25 and 35, and must possess a certificate of not less than three years' training in medical and surgical nursing in a civil hospital of not less than 100 beds which is recognised by the Advisory Board. Full particulars of the conditions of service, and forms of application for admission, may be obtained either by writing to the Secretary at the War Office, or personally from the Matron-in-Chief, at 68 Victoria Street, Westminster. The Matron-in-Chief will see candidates on Tuesdays and Thursdays between the hours of 10 a.m. and 1 p.m.

### IMPERIAL MILITARY NURSING SERVICE.

We are officially informed that Miss A. A. Mackenzie has received an appointment as staff nurse in Queen Alexandra's Imperial Military Nursing Service. Miss G. M. Richards, matron, has been transferred to the Military Hospital, Portsmouth, from the Military Hospital, Devonport; and Miss E. C. Stewart, sister, to the Military Hospital, Wynberg, Cape Colony, from the Military Hospital, Pretoria. Miss V. L. Batteson, staff nurse, has been appointed to the Military Hospital, Hounslow; and the appointment of Miss C. M. Williams as staff nurse has been confirmed.

### ST. GEORGE'S HOSPITAL NURSES AND THE PENSION FUND.

On Friday evening last Mr. Louis Dick, Secretary of the Royal National Pension Fund for Nurses, addressed the nurses of St. George's Hospital in the board-room on the advantages to be derived from joining the Fund. There was a large attendance of the nurses, and much interest was evinced. The chair was taken by Mr. Warrington Haward, F.R.C.S., Chairman of the Committee on Nursing of the Hospital, and there were present the matron, Miss Smedley, and the secretary, Mr. W. H. Davenport. The attention of the nurses was specially called to the fact that in taking out policies for pensions the amount of premium paid was, after two years, returnable with interest at 2½ per cent.; that premiums could be paid monthly, quarterly, or yearly; that in addition to the pension entered for nurses would be entitled to a bonus addition to the amount for which they had subscribed; and that for a small additional premium a nurse could receive sick pay during illness.

### THE LADIES' ASSOCIATION OF GUY'S HOSPITAL.

The annual meeting of the Ladies' Association of Guy's Hospital was held on Tuesday afternoon in the Court-room of the hospital. The Countess of Bective took the chair, and reports of previous meetings were read by Mrs. Shaw, the secretary. The room was well filled by the representatives of the various branches and other ladies also interested in the hospital were present. In the adjoining board-room the garments made during the past year were on view, being grouped according to the branch from which they were derived and the ward for which they were intended. Miss Swift, the matron, said that the work of the Association was immensely appreciated, and that the wards benefited greatly by the beautifully made and useful clothes, and also that similar organisations were being started abroad where Guy's sisters and nurses had gone to take up work. Tea was served afterwards, and many of the ladies visited the wards and other parts of the hospital.

### WESTMINSTER HOSPITAL LADIES' ASSOCIATION.

At the annual meeting of the Westminster Hospital Ladies' Association on Monday, Katherine Duchess of Westminster in the chair, the hon. secretary read the annual report, which showed that 85 ladies had joined during the year, and that the number of members on December 31 last was 155. The subscriptions have increased to the extent of £58 over the previous year.

### THE MOTHERS' UNION AND NURSES.

A great effort is being made by the Mothers' Union to enlist the co-operation of nurses. In a preliminary report of a sub-committee on this branch of the work just issued, it is stated that since the drawing-room meeting in December last at Camelford House, which was addressed by Miss Böge, superintendent of the Nurses' Training Home at Shoreditch, meetings have been held at Guy's and Westminster Hospitals, the Fulham Hospital for Women, the East-end Lying-in Hospital, the Plaistow Maternity Hospital, Chelsea Poor-law Infirmary, the Central Home of the Royal Maternity Charity, and the Samaritan Free Hospital. The movement has the support of Miss Amy Hughes, and several Queen's nurses have attended meetings on behalf of it. Lately, it was decided to try and obtain, by the aid of the matrons, a hearing for the Mothers' Union at several of the general hospitals, with the object not so much for enrolling nurses as members of the organisation as to acquaint them with its work and enlist their sympathy. It is felt that nurses, more especially district nurses, can do much to uphold the sanctity of marriage, and to awaken in mothers of all classes a sense of their great responsibility in the training of the boys and girls, the future fathers and mothers of our Empire. These are two of the chief objects of the Union. Details can be obtained from the special correspondent for nurses, Mrs. Russell, St. Stephen's Vicarage, Battersea Park, S.W.

### PROMOTIONS AT CAMBERWELL INFIRMARY.

As a result of the examination of probationers at Camberwell Poor-law Infirmary, Miss A. B. Carver —who obtained the highest number of marks—and Miss J. Burgess, have been appointed sisters. Nurses J. Stavert, M. T. Wilson, R. H. Blake, E. H. Backhouse, M. E. White, M. Gwinnette have been appointed staff nurses. The Guardians have also appointed Miss L. Creemer to be home sister and assistant matron. She was trained at Camberwell Infirmary, was afterwards ward sister, and is at present doing private nursing in South Wales. We are informed that Miss A. Dyer, trained at Kensington Infirmary; Miss L. Lightowler, trained at Clayton Infirmary, Bradford; Miss G. Pritchard, trained at Stafford General Infirmary; Miss Ethel Sutton, trained at Stapleton Workhouse Infirmary; Miss F. Berry, trained at Islington Infirmary, and recently staff nurse at the North West London Hospital, have been appointed staff nurses.

### NEW NURSES' CLUB.

There has just been opened at 51 Norfolk Square, London, a residence and club for trained nurses, masseuses, and lady medical students. The situation is convenient, and the house itself seems excellently adapted for the purpose. Miss Louise Basan, the lady superintendent, and Miss L. M. Gordon, the secretary, are both trained nurses, and both served for two years in South Africa during the war. Miss Basan possesses, in addition, the Central Midwives Board certificate. The club is intended for both resident and non-resident members. The residents pay an entrance fee of half a guinea and a yearly subscription of the same amount; the rent of their bedroom is 4s. a week, including the use of a wardrobe, box room, and telephone. Food is charged for per meal. Non-residents pay a guinea entrance fee and a guinea subscription, which covers the use of the club sitting-room and dining-room. The drawing-room on the first floor is reserved for residents. The house has been renovated from top to bottom, and rendered as bright and attractive as artistic papers and dainty hangings can make it. There is accommodation for 30 residents.

### MISSIONARY LEAGUE.

The fourth annual conference of the Nurses' Missionary League will take place next Wednesday at University Hall, Gordon Square, the three sittings being respectively at 9.45 a.m., 3.30 and 7 p.m. At the morning meeting Dr. Wilhelmina Eger, of Multan, India, will speak on "The Work of Doctor and Nurse in a Medical Mission Hospital." In the afternoon the proceedings will assume the character of a conversazione, the hostesses being Lady Wingate and Miss Oldham. Several workers in the foreign Mission field will narrate their experiences, which, it is expected, will be of much interest to nurses. Mr. McAdam Eccles, F.R.C.S., will preside at the evening gathering, addresses being also given by missionaries from China and Korea.

### THE NURSES OF HOLBORN UNION INFIRMARY.

The medical officer and matron of Holborn Poor-law Infirmary are to be congratulated upon the report of Dr. J. J. Perkins, who recently held an examination of ten probationers and recommended that they should all be granted certificates. The maximum number of votes was 200, and the awards were as follows: Miss Lubke 188 marks, Miss Underwood 184, Miss Barrett 176, Miss Cornish 167, Miss Franks 166, Miss Bond and Miss Judge 162, Miss Price 154, Miss Bullinaria 151, and Miss Kitney 150. The examiner stated that the answers given to his questions showed, almost without exception, very full, accurate knowledge, and also that the class had received very thorough and painstaking teaching. He also noted that the probationers' powers of observation in the wards had been admirably cultivated.

### FOR THE EAST LONDON NURSING SOCIETY.

There were a number of pretty exhibits, some possessing great merits, at the exhibition and sale of water-colour drawings held at 16 Mansfield Street, last week, in aid of the East London Nursing Society. One sympathetic picture of a yellow cat, appropriately named "Ginger," had a pathetic interest, having been painted by an invalid lady who spends all her time upon her back. The pictures being all gifts, of which Lady Maxwell Lyte presented 100, they were marked at quite moderate prices, and we hope that Lady Portsmouth's kindness has resulted in a substantial addition to the Society's funds. All the nurses attached to the organisation were invited to see the show.

### SHORT ITEMS.

On Monday Mrs. Charles Clay was introduced into the King's presence and received the decoration of the Royal Red Cross from his Majesty.—One of the two selected candidates for the post of assistant matron at Crediton Workhouse last week was Miss Bessie Burridge, who as a child was an inmate of the institution.

### The Nursing Outlook.

"From magnanimity, all fears above;
From nobler recompense, above applause,
Which owes to man's short outlook all its charm."

### SOME MATRONS' DIFFICULTIES.

In our two previous articles on "Hospital Housekeeping" and "Faulty Committees" we pointed out the direction in which weakness in administration is often apparent, and indicated the causes of the inefficiency which sometimes exists in regard to discipline in county hospitals especially. These articles have evidently fulfilled a useful purpose, if we may judge from the correspondence which has reached us. We are assured that the difficulties in question are met with by matrons both in the North and South of England, and that they are very similar wherever they occur. Every matron who is fit for her post will gladly state the reasons to her committee for her action in relation to members of the nursing staff, should they be asked for. Such inquiries are welcomed by the ablest matrons, because they exhibit the deep interest taken in her work and department. When the committee is efficient, as we have pointed out already, it is sure to contain some men of business who, from the interest they have taken in the hospital for years, have kept themselves in touch with the working of each department, and are so acquainted with the progress made and the requirements of each. Where both the committee and the matron, or lady superintendent, are efficient, harmonious working is well assured.

But where there is a faulty committee without a capable chairman or business members of standing, the difficulties of the head of the female and nursing staff are well-nigh insurmountable. These difficulties arise from the fact that in these cases the committee exhibits little or no discipline itself, and so individual members of the committee who may happen to reside near the hospital, or who, having abundance of time, are constantly within its walls, though they may not possess any aptitude for administration, are apt to cause trouble. This arises from want of apprehension of what is due to the head of a large establishment, and such ill informed memers of a committee may, and as a matter of fact do, interview private members of the nursing or domestic staff whenever they can learn that it is possible such members may have a grievance. Proceedings of this kind are highly improper on the part of members of a committee, and the regulations ought to render it impossible that such acts of indiscipline shall occur, for they do not in fact occur, in any well administered hospital anywhere.

In order to be efficient the lady superintendent or matron, if she is fit for her position, must have devoted years of hard and careful work and study to enable her to master the details of every branch of administration for which she is responsible. Once having attained her knowledge and training, the more efficient she is the more properly and strongly will she resent interference on the part of less well informed people who may happen by accident to be members of the committee of an institution in which she may be elected to fill the chief post. It is bad enough for a committee to be so careless as to elect a young and recently qualified man to take the post of master of the household in a large hospital; but it is infinitely worse, if the interests of the patients, the public and the profession are considered, to say nothing of efficiency, for a hospital to be run by a committee which has so little regard for its own dignity and responsibility, that it permits the most fussy of its members to interfere as individuals with the general work of a great institution or any of its officers. These things should not be. We hope wherever they do occur that steps may be taken to put a stop to every abuse of the kind.

It is the duty of the subscribers to see that the committee of every institution which they support includes a reasonable number of business men who will undertake to attend the meetings and look after the business. Efficient members of this type should charge themselves with the duty of seeing that the chair is occupied by a gentleman of administrative capacity and sound judgment. The position of chairman of a hospital is a post of great honour. We hope that one effect of these articles will be to make every chairman of a hospital look into the matters we have dealt with during the last few weeks. After conference with his matron, let him next take steps to secure that the regulations of the hospital are so drawn as to remove any possibility of a continuance of the evils which we have so frankly pointed out. Each chairman and every member of the committee must surely be desirous to make the administration of their hospital efficient. Taking this for granted how is it that so many committees leave the matron without intelligent support in her valiant struggles to keep her training school at least from retrogression when she finds it impossible, from the fussy interference of ignorant people, to make continuous progress in the direction of a maximum efficiency? We do not suppose there is a single committee where it is not known to a few of the members that the evils complained of exist, and the causes from which they spring. Why then are they so silent under a system which must make for inefficiency and so imperil their individual credit as trustees for the governors, the patients, and the public? In such circumstances it is the duty of independent members to act independently by supporting the matron and by promptly taking steps to secure the necessary reforms.

### Abdominal Surgery.

By Harold Burrows, M.B., F.R.C.S., Assistant Surgeon to the Seamen's Hospital, Greenwich, and to the Bolingbroke Hospital, Wandsworth Common.

### AFFECTIONS OF THE SMALL INTESTINE.

The small intestine, which is about twenty feet long in the adult, commences at the pylorus and terminates at the ileo-cæcal valve, where it enters the large intestine. Throughout it is attached to the posterior part of the abdomen by a fold of peritoneum termed the mesentery.

The first portion of the small intestine, about ten inches in length, is termed the duodenum, and the remainder the upper half is described as the jejunum, and the lower half as the ileum. So that material travelling down the alimentary canal, after leaving the stomach, passes along successively the duodenum, jejunum, and ileum, and then enters the large intestine.

Regarding intestinal disorders generally, there are two chief complications which demand surgical treatment; these are peritonitis and obstruction.

### Peritonitis.

The subject of peritonitis has been dealt with in a previous article, where it was pointed out that the contents of the small bowel are very septic. If through injury or disease of the intestinal wall this septic matter escapes into the general abdominal cavity peritonitis is sure to result.

The lesions which produce peritonitis in this way are rupture of the intestine from severe blows or crushes of the abdomen, stab wounds and bullet wounds, perforation of the bowel by an ulcer, and gangrene of the bowel, as occurs, for instance, in neglected cases of strangulated hernia. The treatment of acute septic peritonitis has been discussed in the article referred to above, and no repetition is needed except to emphasise once more the fact that early operation is the only treatment that offers any material hope of saving the patient; every hour of delay may cost his life.

Reference has been made also to the fact that in cases of rupture or wounds of the intestine there may be no evidence of grave injury for some time, perhaps a few hours, after the accident. For this reason, in all doubtful cases the patient should be kept at rest on his back under careful observation, until sufficient time has elapsed to clear away all doubt.

Gangrene of the bowel was discussed under the heading of strangulated hernia.

It remains to consider briefly certain special kinds of ulceration of the small intestine which may perforate the bowel walls. These are duodenal ulcer, tuberculous ulcer, and typhoid ulcer.

### Duodenal Ulcer.

Ulceration of the duodenum occurs chiefly in young men between the ages of twenty and forty. Usually the ulcer is within four inches of the pylorus.

The symptoms, complications, and treatment are practically the same as in cases of gastric ulcer. Not infrequently one of the two chief complications —hæmorrhage and perforation—may be the first manifestation of the lesion.

There are certain points about bleeding from a duodenal ulcer which are worth mentioning. In hæmorrhage from a gastric ulcer the patient frequently vomits the blood (hæmatemesis) and some altered blood may be passed in the stools, which appear black in consequence (melæna). But in hæmorrhage from a duodenal ulcer the patient does not vomit the blood; it all passes away by the bowel in the form of black stools. Consequently, the bleeding is easily overlooked, the more so because, as stated above, there may be no other evidence to suggest the presence of a duodenal ulcer. Anæmia suddenly developing in a young man, especially if it is associated with faintness, should lead to a suspicion of internal hæmorrhage, and accordingly a careful watch should be maintained for any appearance of melæna. Indeed, in all intestinal cases it is essential to keep a look out for any abnormality of the stools, and if they present any unusual appearance, they should be kept for the medical attendant to examine.

### Tuberculous Ulcer.

Ulceration of the ileum is an occasional complication of pulmonary tuberculosis, though the lesions in the bowel may give rise to no definite and recognisable symptoms. Persistent or frequently recurring diarrhœa associated with colicky pains are the chief evidences. Hæmorrhage and perforation may be caused by the ulcers. The symptoms and treatment of perforation of a tuberculous ulcer are the same as in the case of perforation of an ulcer in the course of typhoid fever.

### Typhoid Ulcer.

In typhoid fever numerous ulcers occur in the lower part of the small intestine. Like other ulcers in the intestinal tract these may open up a blood-vessel and cause hæmorrhage, or may perforate the bowel wall and produce septic peritonitis. Hæmorrhage from a typhoid ulcer is not amenable to surgical treatment, but in the case of perforation early operation gives the patient his only appreciable chance of recovery. But there must be no delay, and so it is necessary that the nurse who is constantly with the patient should be able to recognise at once any symptoms which are at all suggestive of this untoward complication.

Perforation, like hæmorrhage, seldom occurs in typhoid fever until the third week of the illness. It is usually accompanied by severe pain in the abdomen and symptoms of collapse. The patient's pulse becomes more frequent and smaller in volume, while his temperature falls, his face becomes cold and soon shows the "abdominal expression," very likely he vomits, and his abdomen becomes rigid, tender, and distended. These symptoms do not all develop instantaneously. The first to appear is pain in the abdomen, and if a patient who is suffering from typhoid fever complains of severe abdominal pain, this should be sufficient to arouse grave apprehension. If the pain is followed by any of the other symptoms just mentioned, such as a marked fall of temperature, or an alteration in the frequency or character of the pulse, energetic preparations must be made for the treatment of a perforated ulcer.

In no case ought the nurse to take upon herself the responsibility for any delay in these cases. It should be a rule in nursing a case of typhoid fever to note down on a piece of paper any sudden complaint of abdominal pain, or any sudden alteration of pulse or temperature, and immediately despatch the information written down in this way to the medical attendant.

Of other perforations of the bowel there is little to be said. The symptoms are much the same in all cases, namely those of a sudden acute inflammation of the peritoneum. Most comments which have been made already in connection with septic peritonitis and perforated typhoid ulcer are applicable to perforations of the intestine from any cause.

To sumarise the duties of a nurse in these cases, they are: (1) to recognise at once the necessity for obtaining medical assistance; (2) to write down on a piece of paper the symptoms which give rise to apprehension, such as abdominal pain, vomiting, or tenderness and rigidity of the abdomen, including in her statement the patient's pulse-rate and temperature; (3) to counteract shock by applying hot bottles to the patient's extremities; (4) to withhold all food by the mouth; (5) to see that preparations are made for an immediate operation in case it may be necessary.

The object of an operation is to close the perforation with sutures, to cleanse the peritoneum in the neighbourhood of the perforation, and by means of drainage tubes to allow any subsequent inflammatory effusion to escape.

### The Nurse's Clinic.

### HÆMOPTYSIS.

A patient suffering from hæmoptysis (or bleeding from the lungs) should be kept perfectly quiet in a recumbent position in bed; no movement of any kind being permitted—excitement, worries of any sort being especially hurtful. No visitors should be allowed to enter the sick-room—in fact, no one, except the doctor and the nurse should be admitted, so long as the hæmorrhage persists. Talking should not be allowed; it generally means a fit of coughing as its sequel, which opens afresh the diseased portion of the lung, and more hæmorrhage is the result.

Tell your patient not to be frightened, as many people are much alarmed at the sight of blood, especially when in a low, nervous condition. Therefore be reassuring and comforting. Do not bustle about: bustle is very infectious. A calm nurse makes a calm patient.

Give nothing hot to drink. Keep your patient upon light diet. A cold compress over the affected area relieves pressure, and ice is soothing to suck. Drugs I shall not touch on. The medical attendant will advise as to those. But if you are nursing a case of phthisis, and hæmorrhage should set in before medical aid can be procured, follow these rules and you will not go far wrong:—

1. Put the patient flat in bed, and, if there is any feeling of suffocation, loosen the clothing about throat and chest.
2. If there be thirst, give iced water in sips.
3. Open the window.

Of course, keep your patient warm. He will probably complain of cold. In that case apply a hot-water bottle to his feet.

4. Keep the patient calm and comforted.
5. Do not administer any drugs till the doctor comes, and on no account give stimulants. I know it is a common practice with the untrained and inexperienced to fly for the brandy-bottle, but it cannot be too strongly condemned. Should the patient faint the hæmorrhage will, in all probability, cease. This is often Nature's means of cure.

### Training in Massage.

There is a great boom just now for physical culture, since the excellent report on physical deterioration by the Duke of Devonshire's Committee. It is curious how England takes so slowly to new methods; massage has been recognised for many years in this country as of great service in certain cases, and yet it is only in quite a few hospitals that regular departments have been started. In Sweden and Germany the number of cases recommended for this treatment are enormous, and France is not far behind in this matter.

Our standard of technique at present is not high. With the exception of foreigners coming from the Central Institute in Sweden, the subject is mostly in the hands of nurses. Now, to do massage in a really efficient manner a great amount of actual physical energy is necessary, and therefore a large number of nurses are not fitted for it. Matrons and others who advise them to take it up ought to be very careful in their choice. It would be a great boon to the country if the training was in the hands of those who had been through it abroad, at least till we had got a thorough mastery of the subject. The course in Sweden takes three years, and yet the majority of nurses in England qualified to practise only spend three months at it.

Apart from massage possibly more may be done in our elementary schools for the further development of exercises, clubs, dumb-bells, and the like, not with the insane idea of developing enormous arm and leg muscles, but more particularly towards the improvement of the chest and back, the perfecting of a good figure, and the maintenance of the general health.

A committee has been formed in London which is endeavouring to get something done in this direction, and meetings have been held in Scarborough, Bristol, and other places to forward the movement.

### To Nurses.

We invite contributions from any of our readers, and shall be glad to pay for "Notes on News from the Nursing World," "Incidents in a Nurse's Life," or for articles describing nursing experiences at home or abroad dealing with any nursing question from an original point of view, according to length. The minimum payment is 5s. Contributions on topical subjects are specially welcome. Notices of appointments, letters, entertainments, presentations, and deaths are not paid for, but we are always glad to receive them. All rejected manuscripts are returned in due course, and all payments for manuscripts used are made as early as possible after the beginning of each quarter.

### The Earthquake in San Francisco.

### AN ENGLISH NURSE'S EXPERIENCES.

Thinking that my fellow workers would like to hear direct from San Francisco during this awful disaster, I am going to make the attempt to send an account of some of my experiences there. I am at this moment writing in a room, in a private house, where I am caring for three ladies, two who have become mentally unbalanced from the disaster, and the third injured by falling debris. Although we are in Oakland, a beautiful suburb of the ruined city we are not altogether out of danger as many buildings are in ruins from the earthquake, though the fire did not reach us. For we are constantly experiencing shocks severe enough to make us hold tight to the nearest fixture, and occasionally to make us prepare to get outside in a hurry.

At the time of the earthquake I was about 150 miles south of San Francisco, and staying with a friend who had a very croupy baby. We had gone to bed and were all in a sound sleep when we woke with a start. The beds were rocking to and fro, every bottle on the shelf fell with a smash, and in the kitchen all the cooking utensils came down. The little Japanese cook flew into the room shouting the house was "full of bad devils." About two hours after news came of the terrible disaster to San Francisco, and the need of help. I determined to go up by next train, and meanwhile, when my intention became known, I became inundated with names and addresses of people who had friends and relatives there.

Two of our medical men were in the city at the time, and I hoped to meet them, which by dint of good fortune I succeeded in doing.

### An Emergency Hospital.

It would take a three-volume novel to tell of all my dangers and difficulties. I got to Oakland, and the peaceful town was full of panic-stricken refugees. Soldiers were also on the spot and martial law already established. I went from one place to another, for nothing was yet organised, and "red tape" troubles embarrassed me on every side. At last I saw our two doctors and they assured me it was useless my staying, as well as dangerous, but as I had come this far I persevered, and at last got to the first Methodist church, which is a large building containing not only the church portion, but many lecture-rooms, Sunday-school rooms, etc., etc.; also a huge kitchen. I was shown the rooms set apart for the "hospital," for this building was turned into an emergency hospital, as well as a place for registering the names of those who were saved. Also it contained dining-rooms where hundreds were fed, and rooms where wagon-loads of clothing were coming in constantly to be distributed to the sufferers. As I entered the hospital I saw a young doctor dressing a badly burnt man, and inquired if he wanted a trained nurse. He told me to wait till one of the seniors arrived, but meanwhile to make an old Irish woman take a dose of Epsom salts, which it had appeared she had refused to do, saying that it was "a little hot gin she was craving." He also said I was to examine her for injuries, as she was complaining that a "whole building had fallen on the top of her." I am happy to say that I went through the test all right, perhaps because I had made up my mind to do whatever came to hand, whether nursing or otherwise. I reported that I could find no bruise or injury, and as the woman was soon found to be an impostor and thief, I was "taken on." I am sorry to say that she was by no means the only one, many being so bold that it did not need Sherlock Holmes to discover them. Principally we had this trouble with morphine fiends and chronic drinkers.

### Mutilation by Looters.

There were eight nurses and one other fully trained nurse beside myself. Two of the staff were probationers who had lost everything in the fire. We had four doctors and four medical students, and they were busy night and day. The other trained nurse was called to a case she had been expecting, which left me in sole charge—both night and day. Two days I was sent over to San Francisco, my red-cross badge and yellow relief committee badge giving me free passage, while a specially signed pass passed me through "the lines in San Francisco." I went with two other nurses, one gentlemen, and another lady. We were to search out approaching maternity cases or any person needing special

A Scene in San Francisco.

care, and also to hunt up a girl of nineteen who had been lost in the rush, and whose mother, with seven other children, we had been anxious to send away to friends in Georgia. To do this there were a good many forms to go through; but I received nothing but kindness and courtesy from "Uncle Sam's Boys." Another task I had was to hand in some of the names to the chief information bureau from the friends I had left. As I found I had some time to wait, I sat down on a doorstep, and presently a ladylike woman came to me with her hand tied up; she seemed in great pain. I invited her to sit beside me, and asked about her injury. She told me she had put on her husband's and her own valuable rings to save them when escaping out of their house; that something fell on her, stunning her; and that when she became conscious the two middle fingers of her left hand containing the rings had been chopped off by some brute. She was looking for information of her son who was in the building at the time. I had heard that the soldiers had shot down a number of men looting, who had been found with their pockets full of ears and fingers which they had cut off in their haste to steal jewellery. There was a man shot down at one corner and a paper pinned on him as a warning to others. There were many killed by the soldiers, and much criticism was rife, but I could see that severe measures were necessary, though at times mistakes were undoubtedly made.

### Maternity Patients.

The ruins, which were still smouldering, made me think of the pictures of "Rome burning," and, strangely enough, we saw and heard a man playing on a piano which had been carried out in the road, and dance-music at that! Not 100 feet away the stench from burning bodies underneath the bricks and debris was sickening. In many places this loathsome smell was so strong that we had to put our handkerchiefs to our noses and mouths to get past. We found three

A Private Residence at Oakland.

expectant mothers and a very sick man who needed care, and these we escorted to comfortable quarters in Oakland. I can only leave to the imagination of the reader an idea of the horrors we saw there. One could not help being struck with the bravery of the many, and the way they tried to take everything cheerfully. I asked one philosophical old woman, who was sitting on her trunk, cooking beans on her stove in the middle of the street, how all could be so brave and even cheerful, and she said, "Well, you see, misery loves company, and we are all a brotherhood now; the rich ain't no longer richer than we 'uns, and we know nothin' much worse can come anyways."

### Feeding Starving Babies.

That night we had several deserted babies brought in, five between one and two in the morning, each carried in the arms of a great rough soldier. They were literally starved, and we had to use care in feeding them. That night we were short of lady nurses, but the four undergraduates and I stripped, bathed, fed, and clothed afresh the poor little sobbing mites, and at last they fell asleep; one little blue-eyed golden-haired baby girl, evidently of good parentage, clasped in the arms of a little half-Spanish dusky little waif, but all now on a common level. The next day we had a busy time: we had to spare two of our best nurses for San Francisco and another was ill. About noon one woman arrived amongst a crowd and soon developed violent mania. She had witnessed the death of her father and only son, being slowly burned under debris that could not be removed in time to save them. We heard that in some cases like this the soldiers mercifully put an end to their sufferings. The poor thing was both homicidal and suicidal, and we had at last to use force and have her taken to a suitable place, as it was impossible to care for her near women and children themselves not far from being in her condition; in fact, we had several who were not normal.

That afternoon four soldiers brought in a man with delirium tremens, who gave a good deal of trouble; then a constable brought in another, who was in a very jocular state of mind in between the "trembles." Then a morphine and drink fiend came in, and we searched him, but only found a razor on him, which the doctor confiscated. Towards morning everything was quiet, when a burly constable came in and the doctor had an important call. This only left one medical student, one male nurse, and myself in the hospital. I had risen to take the malted milk round to the hungry babies when something whizzed past me, and stooping down the medical student found a penknife covered with blood. We were not long making an examination, but happily found that the morphine fiend had such a limited knowledge of anatomy that he had cut his throat in the wrong place. However, we bound up the wound, which was bleeding freely, and the constable walked him off to the "cooler," where doubtless he became a "wiser man."

### Working under Martial Law.

It was my first experience working under martial law, but I think that without this provision everything would have been pandemonium. Even as I was closing these remarks, a minor shock, of which there have been many, shook this house, and I had to leave to reassure my poor nerve-racked patients. This recalls to mind an incident that occurred one afternoon in the church upstairs. There were a large number of people in the auditorium, and the head doctor and I were going the rounds of the sick babies, when a terrific shock started. He said "Nurse, go to the other door and guard it." I did just in time, and the women made a rush, when he shouted, "Don't be scared, women, that is only the baggage being brought up and dumped into the top floor. Nurse H. there can tell you there is no need for alarm." Of course I shouted that the doctor was right, and that I was witness to it being so, and thus we prevented panic. Afterwards the doctor said, "I must say, nurse, that I never felt so like making a run of it myself. I was helped by seeing you so calm." I had to acknowledge that the thumping of my heart was almost as audible to me as the crash upstairs (which proved to be part of the steeple and a chimney falling in), and that it really was a great temptation to give way myself, but that his calmness shamed me.

Everything is quieting down now, and after I have seen these cases through I hope to rest awhile. I am glad I was on the spot, but I shall never forget the sights and sounds of the past two weeks. The one bright edging to the cloud was the brave way in which each bore not only their own burdens, but tried to help others, illustrating Shakespeare's words :—

"One touch of Nature makes the whole world kin."

### The Nurses of Salisbury Infirmary.

### INTERVIEW WITH THE MATRON. BY OUR COMMISSIONER.

Midway between the railway station and the Cathedral at Salisbury stands a large square red brick building with numerous small windows running straight across it at perfectly regular intervals. Passers-by can read the quaint legend upon a board facing the road that this is the Salisbury Infirmary "for the Relief of the Sick and Lame Poor from Whatever County Recommended." Otherwise it would be easy to imagine that the ancient edifice with the river rippling beside it was a castellated keep of the olden times, protected by its armed retainers, with its moat and its drawbridge, rather than a modern Palace of Pain. Even inside the idea is fostered, for though in the theatre and the wards one realises at once that the period is the twentieth century, the wide solid oaken staircase and the many quaint passages twisting and turning into every imaginable corner harmonise with the exterior. So also does the matron's garden, with its prim border of old-fashioned flowers, its stiff hedges, and close-cut lawn.

### The Nurses' Home.

Strangely in contrast with the external antiquity of the hospital is the modern appearance of the Nurses' Home, standing quite apart and reached by a covered and asphalted passage, lighted by electric light. The Home was built in 1900, and extra accommodation was then arranged so as to provide for the staff of the Salisbury Nurses' Home, who at that time received their training in the Infirmary. As they train now before they enter the Home—which is an institution for private nurses in no way connected with the Infirmary—there are a few rooms to spare, a probable saving of much expense to the Infirmary in the future. Here every nurse has a separate bedroom, the necessity for this being a point upon which both the Committee and the matron lay much stress. The rooms are furnished with a wardrobe as well as a chest of drawers and all needful accessories, but there are no fireplaces, the entire building being warmed by radiators. In all, there are 44 bedrooms, which include two bright little rooms for invalid nurses. In serious cases, however, the matron generally arranges for the nurses to be warded. The first and the second floors are occupied by the probationers, the night nurses are on the floor above, whilst the sisters' apartments are at the top of the house, but not over the night nurses' rooms. In order to insure perfect freedom from noise, the rooms over the night nurses' quarters are kept empty or only utilised for boxes. On the ground floor is a sitting-room for nurses and another for sisters, a pleasant reading-room well supplied with magazines, and also a small study where any nurse can insure a quiet time. There is a continuous fire-escape from every floor.

### The Nurses' Garden.

Before the matron conducted me back to the hospital itself—where her own sitting-room is situated—she showed me the garden surrounding the home, which, as well as being rich in flowers, is well stocked with all kinds of fruit, the strawberry patch in particular giving promise of good things to come.

"This," said Miss Vezey, "is the nurses' own special property. They are encouraged to work in it, as gardening is a healthful form of recreation, and in return they are allowed to help themselves to all in season, fruit as well as flowers, a privilege which I think they fully appreciate."

"And are they permitted to make use of the river which I notice has steps here leading down to it?"

"Oh, yes, in their off time. The night nurses, on the whole, are able most often to avail themselves of the boat which one of the officers of the Infirmary kindly places at their disposal in the mornings, and there is nothing more restful for them in the really hot weather than boating.

"Then I conclude that here the night nursing is not disliked?"

"No; in the summer the nurses seem glad when it comes to their turn to take night duty."

"How often is that?"

### Alterations in Condition of Training.

"Every three months, whether junior or senior. I am anxious in the future to arrange that new probationers shall not be obliged to take night duty till the end of the first year, but at present with so many new probationers that is impossible. About the end of last year the conditions of training were altered. Up to that time probationers, who were received for two years' training only, had to pay £10 premium, provide their own uniform, and receive no salary. In consequence, the staff of the hospital ran down somewhat. But under the new regulations we experience no difficulty in getting probationers, though our numbers are less than we could wish, because, unfortunately, owing to lack of funds, two wards have had to be closed and there are just now only 75 instead of 120 beds. One more ward will, however, we hope, be opened shortly."

"How is the staff composed?"

"A night superintendent, a housekeeper, six ward sisters, one staff nurse, one theatre nurse, and 18 probationers. Up till now it has been only during the last month of their training that a nurse has had theatre experience, but by and by I hope to give them a longer time in the theatre."

### The Nursing Students.

"All the probationers now here are training then for three years?"

"No; those who had entered before the new regulations came into force have been given the option of completing their third year; then if they wish to do so, they are placed on the same footing as the others. If, however, they prefer to leave at the end of two years the old conditions hold good in every way. Then, we receive a limited number of 'nursing students', equivalent to paying probationers, who contribute £1 1s. a week and are not received for less than three months. We also give the necessary hospital experience during each year to four students belonging to one of the large institutions where they instruct girls as children's nurses, and likewise to two nurses from a special hospital who train their probationers for a year, then send them for twelve months to a general hospital and receive them back for their last year's training."

"At what age do you receive your regular probationers, and what salary do you give them?"

"From twenty-two to thirty-two years of age, though occasionally, if it seems desirable, I make an exception. Probationers serve one month on trial, paying their own travelling expenses. If they enter only for two years, the terms I mentioned before hold good; if for three years, the first year they are entitled to £8, of which £6 is paid in quarterly instalments of 30s., the remaining £2 being payable at the expiration of the third year. The second year's salary is £12—namely £2 each quarter and £2 at the end of the third year. The third year they are paid £14, £3 each quarter and £2 on the termination of their engagement."

"Therefore when a nurse finishes her training she will have £6 in hand?"

"Yes; I may add that material is given for indoor uniform during the three years, and three weeks' annual holiday is allowed. Outdoor uniform is optional."

### Lectures and Experience.

"What about lectures?"

"Medical and surgical lectures are delivered by the staff during the first and second years, and I hold classes to explain to the nurses any points upon which they are hazy. The training in the wards includes the nursing of male patients, surgical and medical; female patients, surgical and medical; children's cases generally and especially diphtheria with tracheotomy, for which we have had a special isolation ward for the past two and a half years. We have, in addition, one open-air patient who spends his time in a tent on the balcony leading off the male medical ward. So that the nurses get a little experience even in cases of consumption.

"Have you any objection to nurses who are Roman Catholics?"

"No; and they are not expected to attend the chapel service, a rule to be followed by all who belong to the Church of England. We are very proud of our little chapel, the decoration of which has recently been greatly improved."

### Conditional Pensions.

"Are you affiliated to the National Pension Fund?"

"No; but there is a pension fund attached to the Infirmary. In 1863 a Mrs. Fowler, by her will, left a sum of money to such nurses as should have served to the satisfaction of the Committee for fifteen years, and should be disabled by age or infirmity. They are then entitled to a (maximum) pension of 10s. per week or £26 a year. But few nurses now remain so long in one hospital."

On my way out the matron showed me the theatre, which is at the top of the building, quite up to date, but badly in need of a lift for patients. At present the porters have to carry the patients to be operated on; but money is being collected specially for this object, and the alterations will probably soon be completed. Next we passed into the chapel, decorated with lovely spring flowers for Easter, where the chaplain holds daily service, and then through the large bright wards into the entrance-hall.

### An Institutional Badge.

As I said good-day to the matron she drew my attention to the decoration she was wearing. It was a medal containing in the centre the arms of Salisbury surrounded by the words "The sick and needy shall not always be forgotten," and hung from a bar upon which is printed "Salisbury Infirmary." One of these badges has to be worn by every nurse in the institution. That belonging to the matron and the night superintendent is of blue and silver, whilst the sisters wear silver, and the probationers bronze. The badges are given to the nurses and become their property on leaving. They are worn always by the nurses when in uniform. Thus the citizens of Salisbury have no difficulty in recognising the nurses who serve in their Infirmary.

### Presentations.

Queen Victoria's Jubilee Institute for Nurses.—Miss E. Myers, who has been appointed superintendent of Queen's Nurses at Sheffield, and as district assistant has done excellent service for ten years in training the nurses for the Scottish branch, has been presented by the nurses with a gold chain and a writing bureau, bearing the inscription: "To Miss Myers, with affection and esteem, from the Queen's Nurses in Scotland."

### Where to Go.

Southend, Margate, and Ramsgate.—From the Old Swan Pier, London, Saturday, June 2, 9 a.m. By the New Palace Steamers, *Royal Sovereign* and *Koh-i-noor*.

### The Training and Supply of Midwives.

### A QUESTION OF NATIONAL IMPORTANCE.

A meeting in support of the Association for Promoting the Training and Supply of Midwives was held, by permission of Lord and Lady Brassey, at 24 Park Lane, on the 17th inst. In the absence of Lord Balfour of Burleigh, the chair was taken by Mr. Wallace Bruce, the principal speakers being the Mayor of Huddersfield, Mr. Benjamin Broadbent, Dr. Champneys, the Rev. Simeon Singer, President of the Jewish Ministers' Union, and Mrs. Charles Trevelyan. The beautiful hall was well filled, though, as one speaker remarked, in proportion to the needs of the work with which they were concerned they ought to be able to fill the Albert Hall. A letter was read from the Archbishop of Canterbury expressing his sincere regret that he was unable to be present, and his earnest wishes for the welfare and success of the Association.

Mr. Benjamin Broadbent gave an interesting account of the successful working of his own scheme in Huddersfield, where hundreds of infant lives have been saved through a sympathetic and helpful interest shown towards the mothers. He spoke pathetically of the beauty of home-life, of motherhood, especially among the poor, where it is not complicated by conventionality.

Mrs. Charles Trevelyan, addressing herself specially to the mothers present, pleaded eloquently for the needs of a Society which could not fail to interest them, in that it ministered to other less fortunate women in the hour of need that was common to all mothers.

Dr. Champneys, dealing, as he said, with the medical side of the question, spoke strongly of the national importance of the work undertaken by the Association. We were beginning to wake up to the fact that there were not too many children born. For the progress and continuation of any nation it was necessary to keep up an adequate population. The maintenance of this population was a State affair, and should be taken seriously in hand. It was no use talking about an adequate birth-rate if we did not see to it that the mothers were so tended that they were restored to health to all mothers. Every safe and successful birth was an and the capacity to bear more children, and that the children, having arrived here, were enabled to stay and grow up to propagate a healthy race, instead of dying in infancy. We had to recognise that at the gate of life stands the midwife. To her belongs the power of warding off those dangers to the mother and child which, in our present state of civilisation, were so terribly to the fore. He would instance one only—ophthalmia. People did not realise the enormous amount of blindness due to this disease—the result of carelessness and ignorance—alone. It was a serious thought that in spite of the tremendous advance in medicine and surgery, the mortality in child-birth was practically as high as it was thirty years ago. This Society was doing a great work, and if properly supported and enabled to send out an adequate supply of skilled midwives into the country he believed the rate of mortality would steadily decrease and the stain upon the nation be wiped off.

Miss Lucy Robinson, Chairman of the Training Sub-Committee, speaking as a midwife with practical experience, added her testimony to the need of such work. Mrs. Charles Ebden, member of the Executive Council, gave a brief summary of the work of the Association during the past year. Finally, the Rev. Simeon Singer moved a resolution commending the Association to the support of the public as contributing largely to the national health, which was unanimously carried, and the proceedings closed with a vote of thanks to Lord and Lady Brassey.

### Sarah Gamp v. the Modern Nurse.

MISS GENN'S ONSLAUGHT.

By the kind invitation of the Queen Square Club a number of members of various nursing institutions were privileged, on May 16, to hear a lecture by Miss Genn, which was naturally of the greatest interest to them, though they were far from being in sympathy with the ideas of the clever and ingenious lecturer. Her subject was, "That Mrs. Gamp was Preferable to the Modern Trained Nurse." The speaker was, however, less eloquent on the graces and virtues of Mrs. Gamp than she was on the iniquities and shortcomings of the modern nurse. The latter was described as being in many cases conceited, pert, inconsiderate, untruthful, and tactless. Her chief idea while going through the hardening process of three years in hospital was to capture and inveigle into matrimony any raw and youthful medical student who was sufficiently susceptible to her charms! Mrs. Gamp certainly was untruthful, but her lies were so obvious that they could hardly count as lies. She also had the unpleasant habit of peppering snuff into her patient's broth, but this was held to be of no account when set against her conversational charm, flavoured as it was with attic salt. The lecturer was sure that when there was not much the matter, when the patient only needed his forehead bathed and his pillow smoothed, that Mrs. Gamp would suit most people better than the latest product of a modern hospital.

In the debate that followed the lecture various views were expressed. One speaker reminded Miss Genn that Mrs. Gamp did not, according to her biographer, smooth her patient's pillow, but appropriated it entirely to her own use! A doctor thought that the medical student could generally look very well after himself, and that instead of being ruined professionally by marrying a nurse, he was a lucky man if he could persuade her to have him. And a lady, speaking on behalf of an improved and modified Mrs. Gamp, confessed that she would rather have someone to nurse her who would obey her orders, instead of quoting and carrying out the doctor's wishes.

On the whole the audience seemed to think that they were tolerably contented with things as they are. But though the nurses present could not for a moment agree to most of the lecture, yet there were hints here and there which gave them reason to think hard!

We take the following from the *Daily Chronicle*:

A charming and accomplished lecturer, Miss Genn admirably entertained the members of the Queen Square Club last night with a proposition that Mrs. Gamp was preferable to the modern trained nurse.

"The boundless potentialities of the late Mrs. Gamp; her robust humanity, her conversational charm," and, by way of comparison, "the pert, cocksure, tactless person who reigns at present"—these were the chief features of Miss Genn's daring thesis, which she sustained with splendid courage in the presence of a number of uniformed nurses. Here are some of her dicta:

"Choose your specialist, and you choose your disease."

"The typical trained nurse is strikingly deficient"—in most things. She is coarse.

Again: "There is hardly a person in this room who does not know of a case in which a medical student has ruined his profession by accepting the matrimonial proposals of a nurse before realising what he has done. Such cases are little better than kidnapping."

Nurses have, said Miss Genn, attended the wife and afterwards married the widower. "Mrs. Gamp did not marry her patients."

"Take Gamp at her worst, she was still a human being."

"I should be glad of a revival of Mrs. Gamp. In a great many cases all that is wanted is a sensible woman who does not wear creaking shoes."

### The Nurses' Stalls at the King's College Hospital Fete.

The stall presided over by the Sister-Matron and sisters and nurses of King's College Hospital and Mrs. Headlam, at the Elizabethan Fair and Fête in aid of King's College Hospital, which is being held this week, is an octagonal structure standing in the gardens of Lincoln's Inn. There are flowers in immense profusion, fruit, dairy produce, provisions, vegetables, poultry, and even a few live animals. The ladies responsible for the stall cannot speak too warmly of the kind assistance which they have received, both in the way of personal help and in various gifts. From Covent Garden, where are many old patients, came quantities of lovely flowers, while one ex-patient volunteered to come and price all the flowers at the stall. Large stocks of Devonshire cream were presented. The Army and Navy Stores sent a man to set out all the provisions in the best possible way, and to price them. Near the provision stall and the marquee where the theatricals take place is a little row of quaint booths built to imitate the old houses in Holborn. The first of these is in charge of the Countess Roberts and the Army sisters and nurses. One side of the stall is entirely filled with articles made by the soldiers in hospital, and a most interesting, if somewhat pathetic, spectacle it is. Some very good chip carving and poker work trays come from Shorncliffe. In front of the stall is a doll attired as an Army nurse, dressed by a sick officer at Netley, and very well dressed too. There are stockings, jerseys, shawls, scarves, a very nice tea-cosy by "Boy Phillips," tablecloths, cosies, rugs, lampshades, and pictures, hand-painted by a sick soldier. All the work would compare well with what is usually to be seen at ordinary bazaars. The other side of the stall is furnished by the sisters and their friends, and contains some most exquisite piece of work, all marked at very moderate prices.

### Everybody's Opinion.

A FRIEND OF NURSES.

The Senior Nurse of the Neath Nursing Association writes: In your last issue you state that the late Miss Claudia Griffiths left £5,000 to the above Association. May I correct the mistake; it is £500 (hundred). This is the third legacy we have been fortunate enough to have—£2,000 from the late Miss Rowland and £50 from the late Mrs. Margaret Rees.

[We are sorry that the daily press made a mistake in a very important cipher.—Ed. Hospital.]

BABIES' NURSES.

"Sarah Gamp" writes: May I suggest that it strikes me as sad rather than amusing that trained nurses should pour contempt upon babies' nurses? Working privately I see a good deal of the work done by babies' nurses, and I think they have a far more trying life than ours: they are with their little charges day and night, have no "off hours," and often receive small appreciation for their loving care, which includes the first training of the character, besides that of the physique, of our rising generation. Your correspondents claim that there should be some obvious distinction between the uniform of hospital nurses and that of baby's nurses, but if we are so vastly superior, surely our appearance, our manner, and our speech should sufficiently differentiate us from the lower order of untrained (namely, not hospital-trained) women. Moreover, we must confess that our indoor uniform was originally borrowed from the costume of the domestic servant in the middle of last century. Do uneducated men look like gentlemen because they happen to wear the same clothes? Would it not be thought "amusing" if it were proposed that only university graduates should wear black coats?

### THE NURSES' SOCIAL UNION.

Miss F. C. Joseph writes from Woodlands, Holford, Bridgwater: The Nurses' Social Union in Somerset, of which you gave so admirable an account in your issue of April 14, is steadily growing in the county. During April four meetings were held at Weston-Super-Mare, Shepton Mallet, Norton Fitz Warren, and Bath. At Shepton Mallet Miss Ellinor Smith, the superintendent of the County Nursing Association, gave an excellent address on "District Nursing," while the other three meetings had the great pleasure of listening to Mrs. Clare Goslett, who gave them intensely interesting lectures on the "Claims of Hygiene on the Attention of Nurses," to which her audiences listened spellbound. These meetings all presented features of special interest. The Union had not before gained a footing in Weston, but by the kindness of Mrs. Wallace, the wife of one of the leading medical men in town, who acted as hostess, a representative audience was secured and a foundation we hope laid for an extension of the work of the Nurses' Social Union in the future. The meeting for the Taunton branch was held by the kind invitation of Mrs. Wilfred Marshall at her house, Norton Manor, and a fine day enabled the nurses to enjoy the beautiful gardens as well as the lecture and tea. Bath was a new centre, and those interested in the movement had been a little doubtful whether it would succeed in so large a town. But any apprehensions were set at rest by the meeting, which was held by Miss Herringham's invitation at her house in St. James' Square. Over sixty nurses attended from the hospitals, infirmaries, district and private homes, and one and all went away delighted, while many have been the letters received expressing their enjoyment of the afternoon and their desir for another meeting soon. At all these gatherings the little loan collection of nursing appliances has proved of great attraction. The district nurses are glad to have an opportunity of seeing the most up-to-date appliances, while their hospital sisters are not above taking a hint as to how a cradle may be made from a child's hoop, etc., to put into practice, perhaps, at some future day. Most of the heads of the nursing profession in the county have become honorary members of the Union, and Mrs. Clare Goslett, whose interest in it is very great, has also given her name. The Nurses' Social Union is still almost in its infancy, but it is growing fast, and should ere long find a welcome in other counties.

### Appointments.

Ashton-under-Lyne Union.—Miss Annie Etchells has been appointed charge nurse. She was trained at Ashton-under-Lyne Poor-law Infirmary, and has since been charge nurse at Prestwich Poor-law Infirmary.

Basingstoke Cottage Hospital.—Miss Juliana Smythe has been appointed staff nurse. She was trained at Tonbridge Poor-law Infirmary, and has since been night nurse at St. Catherine's Hospital, Ramsgate. Prior to her general training she was assistant nurse on the hospital ships at Long Reach, Dartford.

Brockley Cottage Hospital.—Miss Gertrude Bulton has been appointed nurse matron. She was trained at Wandsworth Poor-law Infirmary, and has since been sister at the Lady Forester Trust Hospital, Much Wenlock.

Chesterfield Union Infirmary.—Mrs. Mary Lisson has been appointed night sister. She was trained at Toxteth Infirmary, Smithdown Road, Liverpool. She has since been sister at the Infirmary, Mill Road, Liverpool, and sister at Highfield Infirmary, Knotty Ash, Liverpool. She holds the certificate of the Central Midwives Board.

City Isolation Hospital, Bagthorpe, Nottingham.—Miss Eleanor Horsfall has been appointed night superintendent. She was trained at the Lying-in Hospital, Liverpool and the Mill Road Infirmary, Liverpool. She has since been theatre charge nurse at the General Infirmary, Hertford, and sister at the Isolation Hospital, Bagthorpe, Nottingham. She holds the certificate of the Central Midwives Board.

Eston Urban District Sanatorium.—Miss Josephine H. W. Whittaker has been appointed matron. She was trained at the North Eastern Hospital for Children, London, the South Devon and East Cornwall Hospital, Plymouth, and the London Fever Hospital. She has since been sister at the London Fever Hospital, the Nottingham Borough Hospital, matron of the Bath Statutory Hospital, and superintendent of the Coronation Nursing Home, Hove, Sussex.

Forest Isolation Hospital, Mansfield.—Miss May Stainthorpe has been appointed Matron. She was trained at the Middlesborough Fever Hospital and the General Infirmary, Huddersfield, and has since been sister of women's and children's wards at Stanley Hospital, Liverpool, sister of medical and surgical wards, and theatre sister at Mill Road Infirmary, Liverpool. She has also done private nursing in Liverpool, and has lately been night superintendent at the City Isolation Hospital, Bagthorpe.

Infectious Hospital, Fraserburgh.—Miss Jessie Taylor has been appointed Matron. She was trained at Aberdeen, and has since been Matron of the Thomas Walker Hospital, Edinburgh.

New Fever Hospital, St. John's, Newfoundland.—Miss C. Duncan has been appointed matron. She was trained at the Meath Hospital, Dublin. She has since been on active service as a member of the Army Nursing Service Reserve in South Africa and at the Cambridge Hospital, Aldershot.

Salop Infirmary.—Miss Holly F. Morrison and Miss Ethel L. Roberts have been appointed sisters. They were both trained at the Salop Infirmary. Miss Morrison has since been staff nurse at Charing Cross Hospital, London, and has done private nursing. Miss Roberts has since been charge nurse at the North-Western Fever Hospital, London, and has also done private nursing.

### Thrift and Insurance for Women

An important conference will be held at Denison House, 296 Vauxhall Bridge Road, S.W., on the above question on Monday, May 28, at 4.30 p.m., which will include papers on "Why Saving is Difficult," "Benefit Societies for Women," "Motives and Methods of Saving," and "Friendly Societies for Women, with Special Consideration of the Sickness Risk from the Actuarial Point of View."

### Novelties for Nurses.

(By Our Shopping Correspondent.)

### COMPO.

Compo is a washing compound after the style of a soap powder. It is made at Messrs. Henry Shaw and Co.'s soap manufactory at Dukinfield. I have caused the sample sent me to be put to the practical test of a laundry expert, and the verdict is that it is the best compound yet tried by her, and that its use in the majority of cases renders boiling unnecessary. We have the testimony of a renowned chemist that there is nothing in Compo to injure the fabrics on which it may be used. In America this compound is highly appreciated. I feel sure that a trial of Compo will insure its constant use.

### PASMA POWDER AND PASMA SOAP.

Pasma Powder is recommended by the makers, Messrs. Curtis and Co., chemists, 48 Baker Street, as the purest and safest toilet and nursery powder. They also state that it was recommended by the late Sir Erasmus Wilson. Personally I have never used a more delightful toilet powder. It is soft and velvety to the touch, and after use it leaves behind it a pleasant smooth feeling. It is not easy to imagine a more suitable application to a baby's tender skin, and the male sex will undoubtedly approve of it for use after shaving. It is also recommended for tender feet. The Pasma soap is a beautifully made and most agreeable soap, especially suitable for use in the case of irritable and rough skins.

### Notes and Queries.

**REGULATIONS.**

**The Editor is always willing to answer in this column, without any fee, all reasonable questions, as soon as possible.**

**But the following rules must be carefully observed.**

**1. Every communication must be accompanied by the name and address of the writer.**

**2. The question must always bear upon nursing, directly or indirectly.**

**If an answer is required by letter a fee of half-a-crown must be enclosed with the note containing the inquiry.**

*Queen's Nurses.*

(109) Is there a branch of Queen Victoria's Jubilee Institution in Dorset?—*Dorset.*

Yes, at Melbury. Dorset has a county arrangement of its own for district nurses.

*Infectious Clothing Regulations.*

(110) Does the corporation, or does the nurse, pay for the return of the clothes she is obliged to leave behind her?—*Bertha.*

We do not quite understand your question. Do you mean clothing at a patient's dwelling, or in a hospital? and do you refer to postage?

*Sick Insurance.*

(111) Is there any advantage in sick insurance for a district nurse? How can it be done?—*M. N.*

All nurses should insure for an annuity and for illness. The Royal National Pension Fund for Nurses, 8 Finsbury Pavement, E.C., insures its policy-holders against sickness. Write to the Secretary.

*Monthly Nurse.*

(112) I am a monthly nurse with surgical experience, but I cannot get into a Home because I have not had three years' training.—*E. S.*

Some Nursing Homes put monthly nurses on their books, though they may not take them on to their regular staff. You had better apply further, and probably you will succeed in getting work in this way.

*Cancer.*

(113) Is it injurious, or would there be danger of infection, by sleeping with a cancer case?—*Enquirer.*

Of course, you mean in the same room. It is not infectious, but it is very unhealthy unless there is a very complete system of ventilation.

*Seamen's Hospitals.*

(114) How can I get a post in a seaman's hospital? How can I become a stewardess?—*A. E.*

Apply to the matrons of the various hospitals. There is a list in "Burdett's Hospitals and Charities," the Scientific Press, Southampton Street, Strand. You can apply to any shipping company, but we advise you not to take such a post. The life is a most trying one, even on the best ships.

*Registration.*

(115) A friend tells me that all my nursing qualifications are useless without registration.—*L. B. C.*

There is no registration of nurses as yet, and your qualifications are sufficient recommendation for any post.

*Nursing in Canada.*

(116) Please give me name of office where I can get addresses of Nursing Homes in Canada.—*J. B.*

Mack Training School, St. Catherine's, Ontario.

*Home for a Child.*

(117) Can you tell me of a home for an illegitimate child of four whose mother has forsaken her?—*Tottenham.*

We are sorry we cannot help you.

*Nurse on a Steamer.*

(118) Can you tell me where to apply as nurse on a ship?—*J. K.*

The only steamship company which employs fully-trained nurses on board ship is the Booth Steamship Company, Liverpool, and the vacancies for such posts are few and far between.

**Handbooks for Nurses.**

| | Post Free. |
|---|---|
| "A Handbook for Nurses." (Dr. J. K. Watson) ... | 5s. 4d. |
| "Nurses' Pronouncing Dictionary of Medical Terms" | 2s. 0d. |
| "Art of Massage." (Creighton Hale.) ... ... ... | 6s. 0d. |
| "Surgical Bandaging and Dressings." (Johnson Smith.) ... ... ... ... ... ... ... | 2s. 0d. |
| "Hints on Tropical Fevers." (Sister Pollard.) ... | 1s. 8d. |

Of all booksellers or of The Scientific Press, Limited, 28 & 29 Southampton Street, Strand, London, W.C.

### For Reading to the Sick.

ON THE ANGELS' GUARDIANSHIP.

Yes, one with us in love
The blessed host above,
And they who wander on a far-off shore;
One in the great Unseen,
Though worlds may roll between,
All join the Holy, Holy, Holy to outpour,
And Christ adore.

O calm and hallow'd hour!
Nor sin nor death hath power,
When that sweet anthem riseth to the throne;
The angels round us stay,
And evil flees away
Before His face, Who came to leave His own
No more alone.

*A. C.*

From childhood upward I should make a custom of saying to a child, "Dear child, thou hast an angel of thine own. When thou prayest, morning and evening, the same angel will be beside thee, will sit by thy little bed clothed in white raiment, will take care of thee, cradle thee to sleep, and guard thee, that the evil one, the devil, may not come near thee. Also when thou sayest the Benedicite and Gratias at meals, thine angel will be at table with thee, serving thee, guarding thee, and watching over thee." If children were taught thus from childhood it would not only make them trust themselves to the guardianship of the dear angels, but it would make them good; for when they were alone they would think, "Although our parents are not with us, the angels are here."—*Luther.*

What a view does this present of the unspeakably watchful and tender care of our Lord and Saviour Jesus Christ, over the very humblest and meanest of His servants! that the whole army of angels and archangels, all the Hosts of the Lord, are set in array for each one of our defence and salvation; ready and glad to refresh us, the members of Christ, as they did Him Who is our Head, after great and sore temptation; to prompt those who wait on us with thoughts for our good, as the angel that spake to Joseph in a dream to strengthen us in agony, as he who appeared to our Lord in the garden; to remove difficulties, and declare good tidings, as he who rolled away the stone, and declared "The Lord is risen." This certainty of angelical aid, so far as we are on Christ's side, we have by His exaltation into heaven, and the subjection to Him of angels, authorities, and powers.—*Keble.*

Those who die in the fear of God and in the faith of Christ do not really taste death; to them there is no death, but only a change of place, a change of state; they pass at once into some new life, with all their powers, all their feelings unchanged; still the same living, thinking, active being which they were here on earth. I say active. Rest they may, rest they will, if they need rest. But what is true rest? Not idleness, but peace of mind.—*Charles Kingsley.*

The sun goes down; the evening shadows lengthen,
The fever and the struggle of the day
Abate and pass away.
Thine angels ministrant, we come to strengthen
And comfort Thee, and crown Thee with the palm,
The silence and the calm.

*Longfellow.*

## Figures and Tables

**Figure f1:**
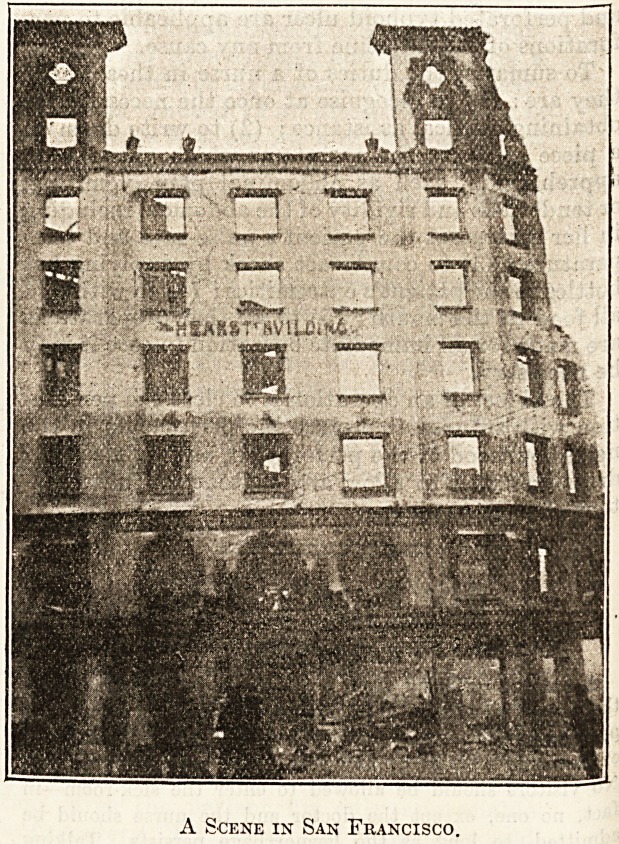


**Figure f2:**